# Cold case: COVID-19-triggered type 1 cryoglobulinemia

**DOI:** 10.1007/s00277-024-05970-y

**Published:** 2024-08-31

**Authors:** L.A.J. (Luuk) van Gils, M.F. (Maarten) Corsten, C.A. (Carin) Koelman, R.J. (Renate) Bosma, R. (Rob) Fijnheer, A.H.L. (Leontine) Mulder, J.C. (Josien) Regelink

**Affiliations:** 1 Meander Medical Center, department of Internal Medicine, Amersfoort, Netherlands; 2 Meander Medical Center, department of Medical Microbiology and Medical Immunology, Amersfoort, Netherlands; 3 Unilabs, Clinical Laboratory, Enschede, Netherlands

**Keywords:** COVID-19, SARS-CoV-2, Viral, Cryoglobulinemia, IgM, Monoclonal

## Abstract

A 42-year-old male was referred to the internal medicine department because of renal failure and persistent malaise after a recent SARS-CoV-2 infection. Blood results showed anemia and severe renal insufficiency (hemoglobin of 10.3 g/dL and a creatinine of 2.19 mg/dL). Additional tests revealed a type I cryoglobulinemia with a cryoprecipitate composed of dual IgM (kappa and lambda). Further investigations on the cryoprecipitate revealed that the immunoglobulins were directed against SARS-CoV-2 antigens. In the meanwhile, our patient noticed improvement of his symptoms accompanied by resolution of laboratory abnormalities. Three months later, the cryoglobulin could no longer be detected.

Type 1 cryoglobulinemia is usually associated with lymphoproliferative disorders and is characterized by various symptoms caused by cryoprecipitates occluding small blood vessels. This is, to our knowledge, the first case of type I cryoglobulinemia with proven precipitation of SARS-CoV-19 antibodies. COVID-19 induced cryoglobulinemia appears to have a mild disease course and to be self-limiting upon viral clearance.

## Background

Cryoglobulins are immunoglobulins (Igs) or a mixture of Igs and complement in serum that precipitate at temperatures below 37 °C and redissolve upon rewarming. Cryoglobulinemia can be classified as type I, II or III based on their composition [1]. In type I cryoglobulinemia, the precipitate is composed of monoclonal Igs, mostly IgG or IgM. Type I cryoglobulinemia is nearly always the result of an underlying B-cell lymphoproliferative disorder - most often Waldenström macroglobulinemia - multiple myeloma or monoclonal gammopathy of undetermined significance (MGUS) [2, 3]. Infection has rarely been reported as the cause of type I cryoglobulinemia [4]. In contrast, type II and III cryoglobulinemia are often related to (viral) infections, particularly hepatitis C virus infection or autoimmune disease. The signs and symptoms seen in type I cryoglobulinemia are related to the vascular occlusion caused by the cryoprecipitate. Cutaneous manifestations like ulcerations, necrosis and/or Raynaud phenomenon are the most common, however peripheral neuropathy, arthritis and renal disease are also frequently encountered.

Since the onset of the coronavirus pandemic in 2019 there have been plentiful reports associating COVID-19 with other diseases, mostly autoimmune disorders such as Guillain-Barré syndrome, systemic lupus erythematosus (SLE) or autoimmune hemolytic anemia [5–8]. However, we are aware of only one prior report of COVID-19 complicated by cryoglobulinemia. We hereby present a patient with COVID-19 and a self-limiting type I cryoglobulinemia with SARS-CoV-2 specific antibodies demonstrated in the cryoprecipitate. We believe it is important that physicians are aware of this rare complication of COVID-19 as it might be under-recognized.

## Case presentation

A 42-year-old Caucasian male with no medical history was referred to the internal medicine department because of renal failure, a normocytic anemia and persistent malaise after a recent SARS-CoV-2 infection. The patient was fully vaccinated against COVID-19, with the last dose administered six months prior to presentation.

Four weeks prior to presentation our patient had experienced a mild episode of COVID-19, as confirmed by reverse-transcription polymerase chain reaction testing (RT-PCR). However, there were persistent complaints of weakness, dyspnea on exertion, anorexia and shivering. History also revealed that shortly after the COVID-19 symptoms started, painless purple spots had appeared on his lower extremities and later on dissolved spontaneously. Based on photographic examination, we diagnosed these skin lesions as vascular purpura. The patient had no history of alcohol consumption and cigarette smoking. Laboratory test results showed anemia (hemoglobin: 10.3 g/dL), thrombocytopenia (90,000/mm3), impaired kidney function (creatinine: 2.19 mg/dL), mildly elevated C-reactive protein (33 mg/L) and a highly elevated erythrocyte sedimentation rate (116 mm/hour). Urinalysis only revealed moderately increased albuminuria with an albumin-to-creatinine ratio of 188 mg/g. Kidney ultrasound showed no abnormalities.

**Fig. 1:**
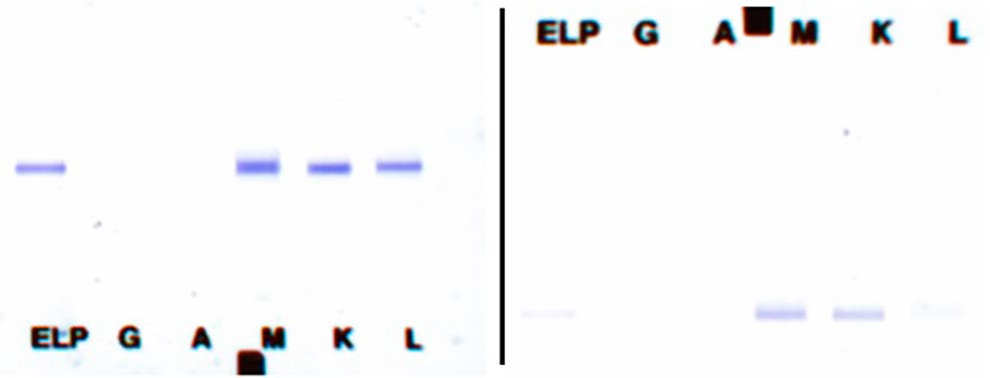
The image on the left shows a immunofixation gel of the cryoprecipitate at diagnosis, clearly showing a dual IgM. On the right an immunofixation gel of the cryoprecipitate 2 weeks later, where the dual IgM is already starting to fade

Additional blood tests aimed at autoimmune disease, multiple myeloma and thrombotic microangiopathies were performed and revealed a type I cryoglobulinemia which was clearly positive, also on repeated testing (Fig. 1). The cryoprecipitate was composed of dual IgM (kappa and lambda) without complement components or rheumatoid factor activity. All other tests came back negative, including ANA, anti-dsDNA, ANCA, complement proteins C3 and C4 and serology for hepatitis B, hepatitis C and HIV.

In search of an underlying lymphoproliferative disorder, that nearly all patients with Type I IgM cryoglobulinemia have, we performed a computed tomography (CT) scan and a bone marrow evaluation including cytomorphology, immunophenotyping and biopsy. However, these investigations failed to demonstrate evidence for clinical lymphoma or a culprit clonal population.

**Fig. 2:**
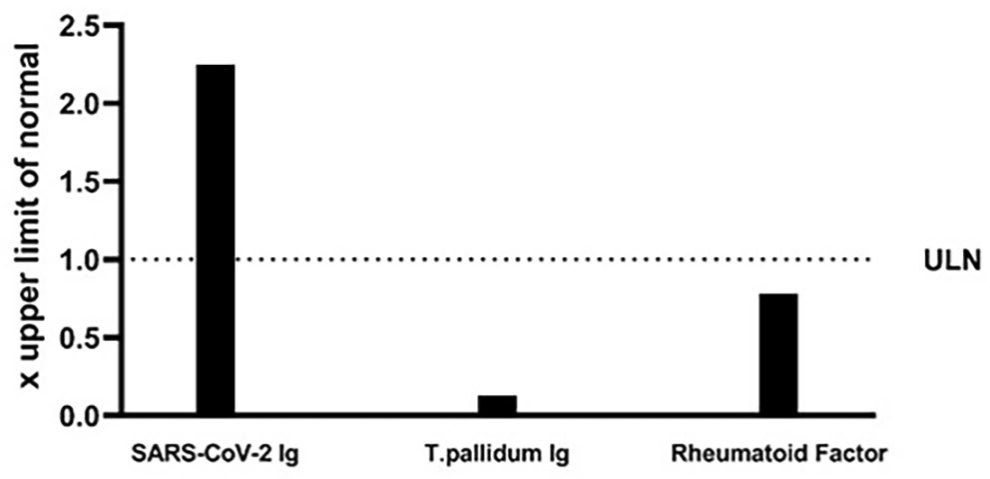
The cryoprecipitate contains SARS-CoV-2 Ig and not Rheumatoid Factor. To check the specificity of the reaction of the cryoprecipitate with the SARS-CoV-2 Spike protein it was also tested for reactivity with T. pallidum antigens. The principle and assay methodology of the T. pallidum Ig assay is similar to the SARS-COV-2 Ig assay and no reactivity of the cryoprecipate was found. To compare the different test results all are expressed as the ratio between the specific test result and the associated upper limit of normal (ULN). A ratio > 1.0 indicates a positive outcome

Because of the absence of a lymphoproliferative disorder and the suspicious time relation between COVID-19 infection and the cryoglobulinemia, we tested the cryoprecipate for the presence of SARS-CoV-2 antibodies. After extensive washing the cryoprecipitate was resolved, and analysis showed that the immunoglobulins in the cryoprecipitate were in fact directed against SARS-CoV-2 Spike protein (Fig. 2).

In the meanwhile, our patient had noticed spontaneous improvement of his symptoms accompanied by resolution of his laboratory abnormalities, and the planned renal biopsy was canceled. Four weeks after the initial presentation his kidney function had completely normalized, and the acute phase reactants had dropped considerably. Lastly, the anemia started to resolve. During follow-up 3 months later, our patient was still doing well and the cryoglobulin could no longer be detected.

## Discussion

To the best of our knowledge, we present the first case of type I cryoglobulinemia with proof that the clonal antibodies in the cryoprecipitate were specifically directed at SARS-CoV-2. One other case of cryoglobulinemia in the setting of COVID-19 has been described, notably also one with a type I cryoglobulin without evidence of an underlying B-cell lymphoproliferative disorder. Of note, this patient also had thrombotic thrombocytopenic purpura (TTP) which required treatment [9]. Additionally, two patients with Sjögren’s syndrome with relapsed type II cryoglobulinemic vasculitis with new onset renal involvement shortly upon SARS-Cov2 mRNA vaccination were described. Also here, no data were available upon the type of cryoglobulin before and after SARS-CoV-2 mRNA vaccination, neither on specificity testing of the cryoprecipitate raising the question whether these cryoglobulins are SARS-Cov-2 specific [10]. Of note, our patient also received SARS-CoV-2 vaccinations but his last dose was 6 months prior to presentation.

Our case highlights the appearance of a type I cryoglobulinemia triggered by COVID-19. The determination of the cryoglobulinemia as type I (i.e., monoclonal) renders this case unique, because cryoglobulinemia secondary to infection is usually a type II or III (i.e., polyclonal). A type I cryoglobulin strongly points towards an underlying B-cell lymphoproliferative disorder, which was not the case in our patient [2]. The exact mechanism by which infection can cause cryoglobulinemia is incompletely understood. Most mechanistic research has been aimed at hepatitis C infection, being responsible for up to 90% of the infection-associated cryoglobulinemias [11, 12]. The leading hypothesis is that chronic hepatitis C infection leads to chronic B cell stimulation and ultimately to the production of monoclonal IgM with strong rheumatoid factor activity against (polyclonal) immunoglobulins directed against the hepatitis C virus core antigen [13]. Chronic B-cell stimulation may be explained by cross-activity against the hepatocyte surface protein CD81 which is used by the hepatitis C virus for hepatocyte internalization but also expressed by B-cells [14]. It is important to note that this mechanism is not sufficient to explain the cryoglobulinemia in our patient, because we found no evidence of a polyclonal component and no rheumatoid factor activity. The immunofixation of the redissolved cryoglobulin of our patient shows a bold monotypic band staining with IgM, Kappa and Lambda (dual IgM light chain). Normally a plasma cell produces only one type of antibody, first IgM and after isotype switching mainly IgG and IgA. These antibodies have either kappa or lambda light chain due to a process of allelic exclusion [15]. This sophisticated receptor editing process enhances the efficiency of B cell development by reducing the number of immature B-cells that undergo apoptosis due to negative selection mainly because autoreactivity [16]. However, B-cells expressing so called dual antibodies have been described [17, 18]. The mechanism leading to dual antibodies has been called allelic inclusion, but the exact mechanism is not elucidated yet. This allelic inclusion might result in survival of autoreactive B-cells [15]. It is not known if and how SARS-CoV-2 infection promotes allelic inclusion. Biclonality cannot entirely be excluded but may be less likely because of the identical migration properties based on charge and size of the protein in the immunofixation and the primary protein selection based on the cryoprecipitation properties. In a case with follicular lymphoma, it is illustrated that two clones with an inverse light chain pattern are using the same Bcl2-IgH recombination, indicating a common progenitor cell [19]. Also, in CLL it is not uncommon that there are bitypic/biclonal presentations. Also here, it is uncertain whether this represents a truly independent biclonal process or whether one clone represents a subclone of the other [20]. In our case, we believe that the cryoglobulin is not a result of malignancy, but probably only a minor part of a fulminant polyclonal response to SARS-CoV-2, whereby the precipitation characteristics leads to protein selection resulting in a type I cryoglobulin presentation (probably due to clonal expansion within a polyclonal response).

Our case illustrates the importance of reconsidering the initial diagnosis when the disease course or results of later investigations fail to match the initial diagnosis. In our case, the type I cryoglobulinemia initially strongly suggested a hematological malignancy. However, the lack of supporting evidence from additional investigations as well as signs of spontaneous recovery prompted us to consider other causes, with COVID-19 being the most likely in our patient. In addition, our case shows that novel diseases such as COVID-19 may present themselves in unexpected ways and therefore we must keep an open mind towards them. Non-invasive testing like characterizing the specificity of the cryoprecipitate might help to recognize COVID-19-triggered cryoglobulinemia.

## Data Availability

No datasets were generated or analysed during the current study.
